# Bulletproof Temporal Bone: A Case of Self-Inflicted Ballistic Injury

**DOI:** 10.7759/cureus.38500

**Published:** 2023-05-03

**Authors:** Maria J Casanova, João T Correia, João Lino, António Magalhães, Luis Meireles

**Affiliations:** 1 Otolaryngology- Head and Neck Surgery, Centro Hospitalar Universitário de Santo António, Porto, PRT

**Keywords:** bullet injury, facial nerve paralysis, cerebro-spinal fluid fistula, temporal bone surgery, temporal bone trauma

## Abstract

Temporal bone injuries due to gunshot wounds are uncommon but devastating, with a high risk of damage to critical neurovascular structures.

The high resistance of the temporal bone, the densest bone in the human body, can sometimes avoid a fatal outcome. However, the complications are in many cases devastating and include hearing loss, facial paralysis, cerebrospinal fluid leakage, intracranial damage, and vascular injuries. Our goal was to report a case of ballistic injury to the temporal bone and describe the surgical approach taken for treatment.

A 74-year-old man was transferred to the emergency room of our tertiary hospital, intubated and sedated, after an attempted suicide with a firearm.

The CT scan showed the metal projectile lodged within the temporal bone on the right side, with the destruction of the ossicular chain and bony labyrinth. After stabilization, sedation was reversed, and the otolaryngology team was called. On examination, the entry wound was located in the cavum concha, with no active bleeding but presenting active otorrhea of cerebrospinal fluid. The patient had complete peripheral facial paralysis on the right side and spontaneous horizontal nystagmus toward the left side. Empirical antibiotic therapy was initiated.

A subtotal petrosectomy was performed, with the removal of the foreign body, repair of the cerebrospinal fluid fistula, obliteration of the cavity with abdominal fat, and closure of the external auditory canal. He was discharged on the 11th-day post-surgery, maintaining complete facial paralysis and right-side anacusis, but was able to walk with assistance.

In conclusion, penetrating trauma of the temporal bone is a potentially life-threatening situation, and patients that survive have a guarded prognosis, as it often leads to permanent sequelae even when managed promptly.

## Introduction

Temporal bone injuries due to ballistic projectiles are uncommon, accounting for 3% of all temporal bone fractures. However, the penetrating trauma caused by these injuries is among the most devastating for vestibulocochlear and facial nerve function [1,2].

The high resistance of the temporal bone, the densest bone in the human body, can sometimes avoid a fatal outcome. However, the complications are in many cases devastating and include hearing loss, facial paralysis, cerebrospinal fluid (CSF) leak, intracranial damage, and vascular injuries [3,4].

## Case presentation

A 74-year-old man was transferred to the emergency room of our tertiary hospital, intubated and sedated, after an attempted suicide with a firearm.

**Figure 1:**
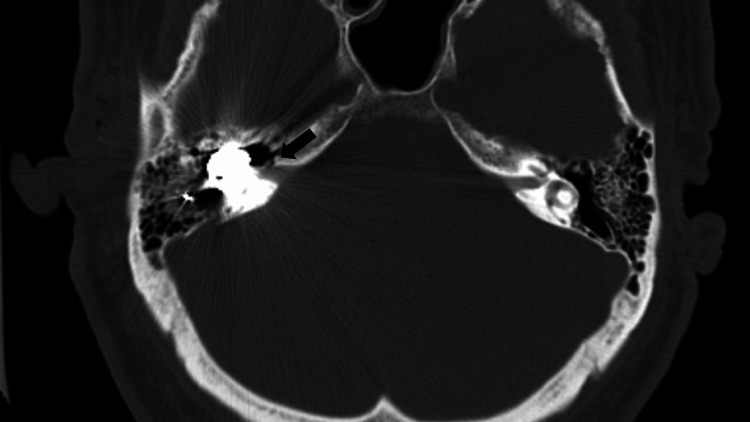
Impacted bullet in ear CT image Axial view of the ear CT scan, showing the foreign body lodged in the middle and inner ear on the right side reaching the most lateral part of the inner auditory canal (black arrow), with destruction of the cochlea and semicircular canals.

The CT scan showed the metal projectile lodged within the temporal bone on the right side, with the destruction of the ossicular chain and bony labyrinth (Figure 1). The ossicles, cochlea, and semicircular canals could not be identified, and the bullet reached the lateral portion of the internal auditory canal. The major vessels were preserved.

After stabilization, sedation was reversed, and the otolaryngology team was called. On examination, the entry wound was located in the cavum concha, with no active bleeding but presenting active otorrhea of the CSF. The patient had complete peripheral facial paralysis on the right side, a degree VI on the House-Brackmann scale, and spontaneous horizontal nystagmus toward the left side. Empirical antibiotic therapy was initiated with ceftriaxone two grams every 12 hours.

**Figure 2:**
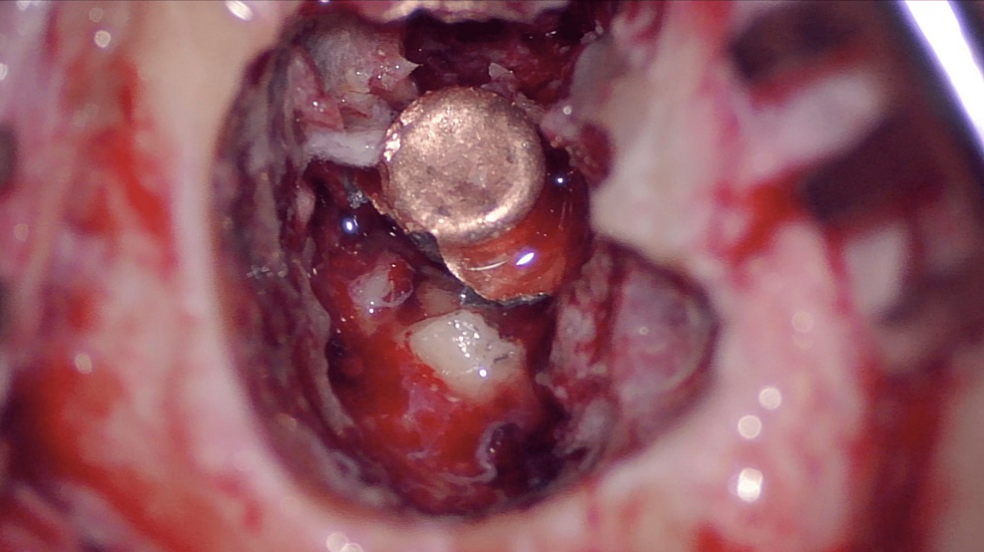
Impacted bullet visualization during surgery Right ear in surgical position: the superior part of the image represents the anterosuperior border of the ear canal, and the inferior right corner of the image is the location of the mastoid tip. The image was taken during mastoidectomy after the removal of bone fragments with complete exposure of the bullet in preparation for foreign body removal. CSF accumulation in the mastoid cavity is visible. CSF: cerebrospinal fluid

The patient was admitted to the operating room 24 hours after arriving at our hospital. After a retroauricular incision, drilling of the mastoid was commenced in order to locate the bullet. Operative findings during mastoidectomy included the destruction of the posterior external auditory canal wall and the absence of the tympanic membrane, ossicular chain, cochlea, and semicircular canals. After the removal of bone and tissue fragments, the foreign body was clearly visible in an image taken during surgery of the bullet impacted within the temporal bone (Figure 2). After the removal of the foreign body, there was a large extension of exposed dura mater in the tegmen and destruction of the medial wall of the cochlea, exposing the internal auditory canal, with a continuous flow of CSF into the cavity. Due to the active CSF leak from this large defect, as well as the complete destruction of the middle and inner ear structures, the decision was made to perform a subtotal petrosectomy, with the closure of the CSF leak and total obliteration of the cavity with abdominal fat in several layers, in order to achieve a minimum risk of intracranial infection. Regarding the facial nerve, during the mastoidectomy, it was found to be sectioned in the mastoid portion, and direct end-to-end coaptation was performed, repositioning the nerve between two layers of abdominal fat. The Eustachian tube was closed using conchal cartilage, temporal muscle, and bone wax. A blind sac closure of the external auditory canal was also performed.

He was discharged on the 11th-day post-surgery, with complete facial paralysis and right-side anacusis, but able to walk with assistance.

## Discussion

According to Portuguese studies, one in every four residents in Portugal owns a firearm, and suicide rates represent 11.7 deaths per 100,000 inhabitants, with men over 75 years old living in rural areas being the most commonly affected [5,6].

Most close-range injuries from ballistic injury have a severe impact on the soft tissues [7]. First-line management includes stabilization of the airway and circulation. After intubating the patient and verifying that there is no major vascular injury, attention can be concentrated on the otoneurological damage.

A systematic review on ballistic injury of the temporal bone has stated hearing loss in 62% of cases, facial nerve weakness in 48%, intracranial injury (stroke, hematoma, infection, carotid cavernous fistula, or carotid artery injury) in 43%, vascular injury in 25%, and CSF leak in 12% [1]. This case presented with profound hearing loss, complete facial nerve paralysis, and a CSF leak.

Indications for early surgical intervention include facial paralysis and CSF leaks [3]. CSF leaks should be managed aggressively due to the risk of developing intracranial infections such as meningitis. In this case, intravenous antibiotics for meningitis were initiated immediately after the identification of the presence of a CSF leak, and surgery was performed 24 hours after the patient’s transfer to our institution. During surgery, bone and squamous epithelium fragments were removed to prevent cholesteatoma; the foreign body was removed to prevent secondary infection; and the cavity was closed using abdominal fat to treat the persistent CSF leak and prevent meningitis [8,9].

## Conclusions

In conclusion, penetrating trauma of the temporal bone is a potentially life-threatening situation, and patients that survive have a guarded prognosis, as it often leads to permanent sequelae even when managed promptly.

In this case, the destruction of the otic capsule and inner ear structures led to peripheral facial palsy, profound hearing loss, and ipsilateral vestibular function loss. The surgery performed, a subtotal petrosectomy, permitted the successful repair of the CSF fistula and the prevention of further complications such as meningitis.
